# The effect of beta-alanine supplementation on neuromuscular fatigue in elderly (55–92 Years): a double-blind randomized study

**DOI:** 10.1186/1550-2783-5-21

**Published:** 2008-11-07

**Authors:** Jeffrey R Stout, B Sue Graves, Abbie E Smith, Michael J Hartman, Joel T Cramer, Travis W Beck, Roger C Harris

**Affiliations:** 1 Department of Health and Exercise Science, University of Oklahoma, Norman, OK, USA; 2 Department of Exercise Science and Health Promotion, Florida Atlantic University, Davie, FL, USA; 3 School of Sport Exercise and Health Sciences, University of Chichester, Chichester, West Sussex, UK

## Abstract

**Background:**

Ageing is associated with a significant reduction in skeletal muscle carnosine which has been linked with a reduction in the buffering capacity of muscle and in theory, may increase the rate of fatigue during exercise. Supplementing beta-alanine has been shown to significantly increase skeletal muscle carnosine. The purpose of this study, therefore, was to examine the effects of ninety days of beta-alanine supplementation on the physical working capacity at the fatigue threshold (PWC_FT_) in elderly men and women.

**Methods:**

Using a double-blind placebo controlled design, twenty-six men (n = 9) and women (n = 17) (age ± SD = 72.8 ± 11.1 yrs) were randomly assigned to either beta-alanine (BA: 800 mg × 3 per day; n = 12; CarnoSyn™) or Placebo (PL; n = 14) group. Before (pre) and after (post) the supplementation period, participants performed a discontinuous cycle ergometry test to determine the PWC_FT_.

**Results:**

Significant increases in PWC_FT _(28.6%) from pre- to post-supplementation were found for the BA treatment group (p < 0.05), but no change was observed with PL treatment. These findings suggest that ninety days of BA supplementation may increase physical working capacity by delaying the onset of neuromuscular fatigue in elderly men and women.

**Conclusion:**

We suggest that BA supplementation, by improving intracellular pH control, improves muscle endurance in the elderly. This, we believe, could have importance in the prevention of falls, and the maintenance of health and independent living in elderly men and women.

## Background

Carnosine (beta-alanyl-L-histidine), a dipeptide is an efficient hydrogen ion (H^+^) buffer over the physiological pH range [1,2]. In muscle, where its concentration is highest, carnosine makes an important contribution to the maintenance of intracellular pH, which is vital for normal muscle function during intense exercise [1]. While the dipeptide is found in both Type I and Type II muscle, its concentration is highest in Type II muscle. Studies in humans and rats have demonstrated an inverse relationship between age and muscle carnosine content [3,4]. Sarcopenia, the loss in muscle mass with age, is associated with significant reductions in strength, power, and the ability to resist fatigue in elderly men and women [5,6]. Significant decreases in skeletal muscle and decline in muscle function are clearly evident after the age of fifty [5,7]. Deterioration of motor coordination, as a result of losses in strength and/or fatigue, is related to an increase in the frequency of falls [6,8] which repeatedly lead to injury and even deaths among the elderly [9].

A number of investigations have used surface electromyographic (EMG) procedures to identify the power output associated with the onset of neuromuscular fatigue (NMF) during cycle ergometry [10-12]. NMF is typically characterized by an increase in the electrical activity of the working muscles over time [11,13]. Moritani et al. [11] suggested that the fatigue-induced increase in EMG amplitude is a result of progressive recruitment of additional motor units (MU) and/or an increase in the firing frequency of MUs that have already been recruited. De Vries et al. [13] developed a submaximal discontinuous cycle ergometer test utilizing this concept of progressive muscle activation and introduced the concept of "the physical working capacity at the fatigue threshold" (PWC_FT_). This utilized the EMG fatigue curves to identify the power output that corresponded to the onset of the NMF threshold. PWC_FT _represents the highest power output that does not result in a significant increase in the electrical activity of the thigh muscle over time (p > 0.05). PWC_FT _has been shown to be reliable [10], valid [10] and sensitive to changes in fitness levels and creatine supplementation [14] in older men and women (62 – 73 years). Furthermore, de Vries et al. [10] suggested that PWC_FT _in elderly men and women may be more appropriate than the assessment of physical work capacity by measurement of maximal oxygen uptake capacity (i.e., V0_2 max_). In addition, de Vries et al. [10] reported PWC_FT _was a more sensitive measure of monitoring changes, in comparison to fitness tests that depend upon heart rate/power output relationships and/or maximal cardiac output.

Recently, Harris et al. [2] and Hill et al. [15] have shown that beta-alanine (BA) supplementation can significantly increase skeletal muscle carnosine levels, and that the increase is correlated to improvements in exercise performance. In addition, Stout et al. [16,17] used the PWC_FT _test to examine the effect of BA supplementation in young (18 to 30 years) men and women, and reported significant increases in PWC_FT _of 12 to 15% with BA supplementation. Accordingly, Stout et al. [16,17] suggested that the increase in PWC_FT _was the result of a BA induced increase in skeletal muscle carnosine, increasing the muscle's capacity to buffer H^+ ^during exercise. However, the effect of BA supplementation on PWC_FT _in elderly men and women is presently unknown. In theory, increasing skeletal muscle carnosine levels, should improve muscle buffering capacity, leading to a delay in fatigue in certain types of exercise. These adaptations may translate into improved functional capacity during daily living tasks and quality of life. The purpose of this study, therefore, was to examine the effects of ninety days of BA supplementation on PWC_FT _in elderly men and women.

## Methods

### Subjects

Twenty-six elderly men and women (Table 1) from independent-living communities in South Florida volunteered to participate in the study. None of the participants had any previous history of BA supplementation and maintained their regular activity and dietary patterns throughout the study. It should be noted; however, that BA is available from meat contained in the diet, although the amount ingested is highly variable according to the meat ingested, and of course quantity. Each participant had a physician's approval to participate in the study, and completed a health history questionnaire prior to partaking. None of the subjects had major orthopedic surgery within the previous year, history of asthma, heart or pulmonary disease, uncontrolled hypertension, and were not taking any medications that would interfere with exercise. All procedures were approved by the Institutional Review Board for Human Subjects. Prior to the initiation of the study, each participant was advised of any possible risks before providing written informed consent.

**Table 1:** Subject characteristics mean (± SD)

	Age (yrs)	Body Mass (kg)	Height (cm)
BA (n = 12)	72.1 ± 10.6	74.2 ± 16.2	159.6 ± 9.6
Placebo (n = 14)	73.4 ± 11.9	73.4 ± 16.5	153.4 ± 10.7

### Experimental design

Immediately following pre-testing, the participants were randomly assigned to one of two treatment conditions using a double-blind placebo controlled design: (a) BA: 2.4 grams of beta-alanine in clear gelatin capsule (CarnoSyn™, Natural Alternatives International, San Marcos, CA, USA), n = 12; or (b) PL: 2.4 grams of microcrystalline cellulose in clear gelatin capsule, n = 14. Each capsule contained 800 mg and was identical in appearance. One capsule was ingested three times per day for ninety days with meals, then within 3 to 5 days participants were post-tested. During the course of the study, the participants were asked to maintain their normal dietary and activity pattern and refrain from starting additional nutritional supplements and nonprescription drugs. In addition, the participants were instructed to refrain from exhaustive physical exercise, caffeine, and alcohol consumption for 24 hours prior to testing. Upon each visit to the testing area, the participants were questioned to assure that they had complied with the investigator's instructions and that they followed the supplementation protocol as directed. Subjects also kept a three-day food diary prior to the pre- and post-testing to ensure caloric intake was consistent.

### Electromyographic (EMG) measurements

A bipolar (2.54 cm center-to-center) surface electrode (Quinton Quick prep silver-silver chloride) arrangement was placed on the right thigh over the lateral portion of the vastus lateralis muscle, midway between the greater trochanter and the lateral condyle of the femur. The reference electrode was placed over the spinous process of the 7^th ^cervical vertebrae. Interelectrode impedance was kept below 5000 ohms by careful abrasion of the skin. The raw EMG signals were pre-amplified (gain: × 1000) using a differential amplifier (EMG100C, Biopac Systems, Inc., Santa Barbara, CA), sampled at 1000 Hz, and stored on a personal computer for off-line analysis. The EMG signals were later bandpass filtered from 10–500 Hz (2^nd ^order Butterworth filter) and expressed as root mean square (rms) amplitude values (uVrms) by software (AcqKnowledge v3.7, Biopac Systems, Inc., Santa Barbara, CA).

### Determination of PWC_FT_

The PWC_FT _values were determined using the EMG amplitude values from the vastus lateralis muscle from the methods previously described by de Vries et al. [13]. The initial work rate for each participant was determined by the principal investigator, based on the participant's estimate of his or her physical fitness. The subjects began pedaling (with toe clips) at 50 rpm on a calibrated, electronically-braked cycle ergometer (Corval 400, Quinton Instruments, Seattle, WA). Power output was increased 10 to 20 W for each two-minute stage of the discontinuous protocol. Rest intervals between bouts were sufficiently long to allow resting heart rate to return within 10 bpm of that obtained upon arrival to the laboratory. During each two-minute bout, six 10-second EMG samples were recorded from the vastus lateralis. The PWC_FT _was determined by averaging the highest power output that resulted in a non-significant (p > 0.05; single-tailed t-test) slope value for the EMG amplitude vs. time relationship and with the lowest power output that resulted in a significant (p ≤ 0.05) slope value [13].

Test-retest reliability for the PWC_FT _test was determined from 11 participants measured 14 days apart. The intraclass correlation coefficient (ICC) was 0.83 (SEM = 8.4 W). No significant difference (p > 0.05) was noted between the mean PWC_FT _values from trial 1 (45.9 ± 17.3 W) to trial 2 (48.3 ± 18.1 W). These results were similar to de Vries et al. [13] who reported no significant (p > 0.05) difference from trial 1 (108.8 ± 53.3 W) and trial 2 (109.1 ± 49.4 W) and an ICC of 0.97 in 16 well-trained elderly men and women (67.6 ± 5.6 years). The difference in mean PWC_FT _values between the current participants versus the de Vries et al. [13] could be due to the mean age difference or trained [13] versus untrained (current study) status of the participants.

### Statistical Analysis

Data were analyzed using 2 × 2 [treatment (BA vs. PL) × time (pre- vs. post)] repeated measures analysis of variance (ANOVA). If a significant interaction or main effect occurred, follow-up analyses using dependent samples t-tests were run. Prior to all statistical analyses, the alpha level was set to p = 0.05 to determine statistical significance. Data were analyzed using SPSS version 12.0 (SPSS Inc., Chicago, IL) software.

## Results

**Figure 1:**
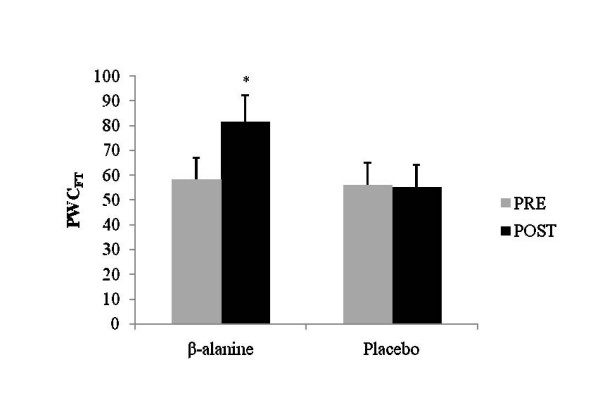
Pre- to post-test values for physical working capacity at fatigue threshold (PWC_FT_) for BA and PL groups. * Indicates a significant difference from pre- to post. (p < 0.01).

**Figure 2:**
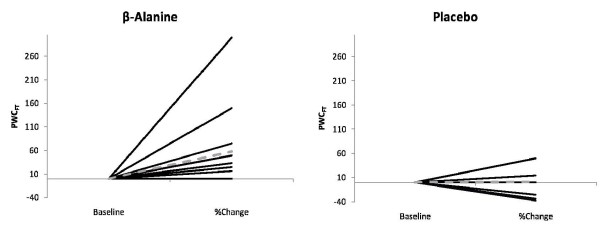
The percent change values for the physical working capacity at fatigue threshold (PWC_FT_) from pre- to post-test for each individual in the BA and placebo treatment groups, respectively. Sixty-seven percent of the individuals in the BA group demonstrated improvements from pre- to post-PWCFT values, compared to 21.5% of the individuals in the placebo group.

A significant interaction was evident between treatment and time for PWC_FT _(p = 0.007). A dependent samples t-test demonstrated an increase in PWC_FT _(28.6%) from pre- to post-supplementation with BA (p < 0.05), but no significant change for the PL group (Figure 1). Furthermore, 67% of the individuals in the BA group demonstrated improvements from pre- to post-PWC_FT _values, compared to 21.5% of the individuals in the PL group (Figure 2).

## Discussion

Data from this study suggest that ninety days of BA supplementation may increase physical working capacity in elderly men and women. These findings may be clinically significant, as a decrease in functional capacity to perform daily living tasks has been associated with an increase in mortality [18], primarily due to increased risk of falls [9]. Further, deVries et al. [13] and Alexander et al. [8] have suggested that falls may be related to fatigue-induced deterioration of motor coordination. Thus, an improved resistance to fatigue, as reported in this study, (Figure 1 and 2) may be important to consider when working with a similar population.

The concept of physical working capacity (PWC), a measure of aerobic power, muscular endurance and efficiency is typically measured by oxygen consumption rate (VO_2_) during a maximal graded exercise test (GXT) [19]. Recently, several studies have reported on the effects of BA supplementation on PWC_FT _during incremental cycle ergometry tests in young men and women [16,17]. Stout et al. [16,17] reported that 28 days of BA supplementation in a younger population (21–27 years) resulted in a significant increase in PWC_FT _by 12 – 15%, respectively. In agreement, the current study demonstrated a 28.5% increase in PWC_FT_after ninety days of BA supplementation. The two-fold increase in PWC_FT _in the elderly compared to young men and women in previous studies may be due to differences in initial skeletal muscle carnosine levels or supplementing duration. In support, Tallon et al. [4] reported that in Type II skeletal muscle, carnosine concentration was 47% lower in elderly (70.4 ± 5.0 yrs) compared to younger (23.8 ± 4.6 yrs) men and women, although Kim [20] found normal muscle carnosine levels in elderly Korean subjects with impaired glucose tolerance. Harris et al. [2] demonstrated that 28 days of BA supplementation significantly (60%) increased skeletal muscle carnosine levels while Hill et al. [15] demonstrated a further 20% increase when BA supplementation was continued for an additional 35 days. In light of these reports, the greater change in PWC_FT _in elderly participants in the present study, compared to previous studies in young men and women, was most likely due to both initial carnosine levels and length of time of BA supplementation.

De Vries et al. [13] suggested the PWC_FT _test may be more appropriate and sensitive to training effects on PWC in elderly, compared to other methods (i.e. VO_2 max_) that require maximal effort and may be ill-advised or possibly hazardous in this population. To our knowledge, this is the first study to examine the effects of BA supplementation using the PWC_FT _test in elderly men and women. However, de Vries et al. [13] did examine the effects of 10 weeks of moderate intensity (70% PWC_FT_) endurance training three times per week on PWC_FT _in elderly men and women (67.9 ± 5.3 years). The results showed a significant (p < 0.05), 30% increase in PWC_FT _as a result of the endurance training. While the results by de Vries et al. [13] support and recommend endurance training as a means to significantly improve physical working capacity, it should be noted that the elderly subjects in the current study were untrained, and the 28.5% increase in PWC_FT _occurred without any type of additional training during the ninety days of supplementation.

It has been proposed that exercise-induced decreases in intramuscular pH may interfere with the excitation-contraction coupling process of skeletal muscle, which, in turn may lead to decreases in power output and fatigue [21]. Maintaining the intracellular pH during exercise could therefore be important for normal muscle function in the elderly [4]. In order to maintain pH homeostasis, various buffering systems are involved, including active H^+ ^export from muscle [2]. However, the immediate line of defense remains the buffering of H^+ ^by intracellular physico-chemical buffers, principally phosphates and carnosine. Marsh et al. [22] demonstrated that there was a significant delay in the onset of intracellular acidosis during progressive exercise after six weeks of moderate intensity training, resulting in an increased capacity for submaximal work in a similar cohort of elderly individuals. Improving the ability to buffer intramuscular H^+ ^accumulation, therefore, appears to be an important factor for delaying the onset of fatigue and increasing exercise capacity in older men and women.

Based on these results, it is not unreasonable to expect that introducing BA supplementation to increase muscle carnosine levels, prior to starting an exercise program in elderly men and women, would lead to an improvement in the quality of training. This would be especially true if muscle carnosine contents were already reduced with elderly persons. The limiting factor for muscle carnosine synthesis is the availability of BA, obtained either from uracil degradation in the liver or from the release of BA from the ingestion of carnosine (and related dipeptides in meat) [2]. From preliminary observations showing that the level of carnosine is reduced by up to 50% in vegetarian subjects [23], compared to age matched controls, it seems likely that the capacity of the body to produce BA is limited and capable of supporting only a limited capacity to synthesize carnosine. This is overcome in meat eaters through the dietary supply of BA. However, elderly subjects, maintained on a diet with a restricted meat intake would be expected to show a similar reduction in their muscle carnosine content as vegetarians, and to respond beneficially to BA supplementation.

## Conclusion

The results of this study suggest that ninety days of BA supplementation may have significantly increased intramuscular carnosine resulting in a 28.5% increase in PWC_FT _due to a greater H^+ ^buffering capacity. However, future studies are needed to confirm our results while measuring changes in skeletal muscle carnosine content.

## Authors' contributions

JS assisted in study coordination, supervision, protocol development and statistical analysis. BG assisted in study coordination, data management and supervision. AS assisted in data management, statistical analysis and manuscript preparation. MH assisted in supervision, data management and study coordination. JC assisted in protocol development and manuscript preparation. TB assisted in manuscript preparation. RH assisted in protocol development and manuscript preparation.
